# Prenatal Hyperhomocysteinemia Induces Glial Activation and Alters Neuroinflammatory Marker Expression in Infant Rat Hippocampus

**DOI:** 10.3390/cells10061536

**Published:** 2021-06-18

**Authors:** Anastasiia D. Shcherbitskaia, Dmitrii S. Vasilev, Yulia P. Milyutina, Natalia L. Tumanova, Anastasiia V. Mikhel, Irina V. Zalozniaia, Alexander V. Arutjunyan

**Affiliations:** 1 D.O. Ott Research Institute of Obstetrics, Gynecology and Reproductology, 199034 St. Petersburg, Russia; milyutina1010@mail.ru (Y.P.M.); anastasia.michel39@gmail.com (A.V.M.); Irinabiolog@rambler.ru (I.V.Z.); alexarutiunjan@gmail.com (A.V.A.); 2 I.M. Sechenov Institute of Evolutionary Physiology and Biochemistry of the Russian Academy of Sciences, 194223 St. Petersburg, Russia; dvasilyev@bk.ru (D.S.V.); tuman-1946@mail.ru (N.L.T.)

**Keywords:** homocysteine, neuroinflammation, glial reaction, hippocampus, neurodegeneration, cytokines

## Abstract

Maternal hyperhomocysteinemia is one of the common complications of pregnancy that causes offspring cognitive deficits during postnatal development. In this study, we investigated the effect of prenatal hyperhomocysteinemia (PHHC) on inflammatory, glial activation, and neuronal cell death markers in the hippocampus of infant rats. Female Wistar rats received L-methionine (0.6 g/kg b.w.) by oral administration during pregnancy. On postnatal days 5 and 20, the offspring’s hippocampus was removed to perform histological and biochemical studies. After PHHC, the offspring exhibited increased brain interleukin-1β and interleukin-6 levels and glial activation, as well as reduced anti-inflammatory interleukin-10 level in the hippocampus. Additionally, the activity of acetylcholinesterase was increased in the hippocampus of the pups. Exposure to PHHC also resulted in the reduced number of neurons and disrupted neuronal ultrastructure. At the same time, no changes in the content and activity of caspase-3 were found in the hippocampus of the pups. In conclusion, our findings support the hypothesis that neuroinflammation and glial activation could be involved in altering the hippocampus cellular composition following PHHC, and these alterations could be associated with cognitive disorders later in life.

## 1. Introduction

Maternal hyperhomocysteinemia (HHC) is one of the common complications of pregnancy that causes various functional impairments of the offspring brain. Studies of the prenatal HHC (PHHC) effect in male rats and mice revealed a decrease in locomotor activity and significant disorders of various types of memory, in particular, in the Morris test [1,2,3,4]. Violation of the reflexes formation [5] and the presence of anxiety was reported in the offspring of rats with PHHC [6], which may cause memory impairment. Our previous data indicate that mature female rats whose mothers had elevated homocysteine (Hcy) level during pregnancy demonstrated disorders of short-term and long-term memory, as well as spatial orientation [7]. At the same time, population-based studies have shown that folate deficiency, high total Hcy and/or low vitamin B_12_ levels in early pregnancy had long-term effects on fetal and child brain development. Thus, the IQ level of six-year-old children whose mothers had Hcy above 9.1 μM during pregnancy was reduced by seven points [8]. In that study, the concentration of the mothers’ Hcy was associated with the level of realization of tasks for language abilities and visual-spatial orientation of their children during the set of neuropsychological tests NEPSY-II [8]. The relevance of additional studies is also emphasized by the fact that the success of vitamin correction of high levels of Hcy, in particular with folic acid, depends on its etiology (vitamin deficiency, lifestyle, mutations in the genes of the methionine cycle enzymes) [9,10,11,12,13]. Therefore, the elucidation of the mechanisms of the consequences of PHHC remains actual and requires further research.

The behavioral abnormalities reported in different studies are not often compared to changes in the cellular composition in the brain structures responsible for cognitive functioning. Studies in rodents have shown that the first month of life is a crucial period for brain development and neuronal maturation, synaptic pruning, cell elimination, myelination, synaptogenesis, and apoptosis [14]. Alterations to the cell functions induced by different pathological factors during this time or earlier may contribute to synaptic abnormalities seen in some neurodevelopmental disorders [15]. Furthermore, adolescence is an important time of neurobehavioral maturation during which limbic structures such as the prefrontal cortex and hippocampus undergo maturation [16]. Previously, we reported that maternal HHC reduced the number of neurons, while activating caspase-3 along with gliosis and increased interleukin-1β (IL-1β) expression and p38 mitogen-activated protein kinase (MAPK) phosphorylation in the cortex of rats on postnatal days 5 and 20 (P5 and P20) suggesting apoptosis and neuroinflammation in this part of brain. However, the specific features of the cellular response to PHHC in different brain regions could be essential and should be characterized and analyzed in detail. Therefore, morphology and ultrastructure, as well as molecular markers of the neuroinflammatory response and cell death were examined in the hippocampus of rat pups. Our hypothesis was that neuroinflammatory reactions are caused by PHHC exposure in the developing brain and can be considered as possible molecular mechanisms underlying the behavioral and cognitive alterations observed by us previously [7].

## 2. Materials and Methods

### 2.1. Experimental Animals

Female Wistar rats were obtained from the Rappolovo Animal Center, St. Petersburg, Russia. The animals were housed at a constant room temperature with a 12-h light and dark cycle and had free access to a 20% (*w/w*) protein commercial chow and clean drinking water throughout the study. Exclusion criteria included signs of illness and behavioral defects at the start of the study. All experimental protocols were performed in accordance with guidelines of the Declaration of Helsinki, and approved by the Institutional Ethics Committees of D.O. Ott Research Institute of Obstetrics, Gynecology and Reproductology (protocol code 88 as of 8 December 2017) and I.M. Sechenov Institute of Evolutionary Physiology and Biochemistry RAS (protocol code 3/2020 as of 18 March 2020).

### 2.2. Chronic Methionine Treatment

**Scheme 1:**
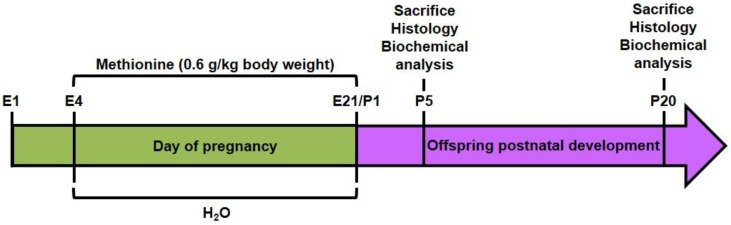
Treatment paradigm followed during the study.

To confirm pregnancy, we verified the presence of sperm in the vaginal smears after mating the female rats. The animals were then daily administrated L-methionine (0.6 g/kg b.w.) in drinking water per os on days 4–21 of pregnancy [17]. Serum Hcy concentration in rats subjected to such treatment achieved levels similar to those described for the serum of patients with mild HHC. Chronic administration of methionine in this dose to pregnant rats caused the increase in the Hcy level after methionine load, not only in the maternal blood, but also in the blood and brain of fetuses, as previously described in details [17]. At the same time, control animals received water. Pups were decapitated at postnatal (P) days 5 and 20. The day of birth was defined as P1. The treatment paradigm followed for the study is illustrated in Scheme 1.

### 2.3. Brain Tissue Preparation for Microscopy

We slightly modified the methodical procedures published by us previously for the brain cortical tissue in the same experimental model [18] to better compare these two brain regions.

On P5 and P20, the hippocampus tissue of pups from control females was compared to that of pups from females treated by methionine during pregnancy (*n* = 10 in each group). The brain tissue blocks were fixed by transcardial perfusion of 4% paraformaldehyde solution in 0.1 M PBS (pH 7.4) with postfixation in the same fixative at 4 °C for a week. Brain tissue was immersed in 20% sucrose solution in PBS (pH 7.4), then frozen and sectioned in the coronal plane using a Cryostat Leica CM 1510S (Leica Microsystems, Wetzlar, Germany). We analyzed 15 µm sections of the dorsal hippocampus in the blocks of brain tissue, starting at the level of Bregma = −4.5 mm [19].

### 2.4. Light Microscopy

Some slices of the dorsal hippocampus were stained by cresyl violet (Nissl) and analyzed using an AF7000 microscope with an DFC495 digital camera (Leica Microsystems, Wetzlar, Germany). The total number of slices analyzed was 10 per animal. The distance between the analyzed slices was 30 µm. CA1 tissue was our main area of interest, as it is known to be involved in the mechanisms of learning and memory formation.

### 2.5. Immunochemistry

We also determined the number of cells labeled by neuronal (NeuN protein) and glial (GFAP, Iba1) marker proteins in the same area of the hippocampus as was specified in the previous section. For this, a sequence of 15 µm sections in frontal plane (10 sections per animal with 60 μm between them) were selected randomly and used for immunolabeling.

The sections were incubated overnight at 37 °C in PBS containing 2% bovine serum albumin, 0.3% Triton X-100 (Merck, Darmstadt, Germany), and one of three antibodies: rabbit polyclonal anti-Fox3/NeuN (ab104224; Abcam, Cambridge, UK; dilution 1:1000), rabbit polyclonal anti-GFAP (glial fibrillary acidic protein, ab7260; Abcam, Cambridge, UK; 1:200), or rabbit anti-Iba1 (ionized calcium-binding adapter molecule, ab178846; Abcam, Cambridge, UK; 1:100) antibody. After thorough rinsing, the sections were incubated for 1 h at 37 °C in florescent-tagged secondary antibodies: FITC-conjugated (ab97050, Abcam, Cambridge, UK; 1:200) or PE-conjugated (ab7007, Abcam, Cambridge, UK; 1:200) secondary antibody against rabbit IgG diluted in the blocking serum. Before mounting, the brain sections were counterstained with Hoechst 33342 (Invitrogen, Waltham, MA, USA) in order to count the total number of cells. Microscopy was performed using a Leica DMR microscope connected to a Leica TCS SL confocal scanner (Leica Microsystems, Darmstadt, Germany). A 488 nm wavelength He/Ar laser was used for excitation of FITC and PE; 350 nm for Hoechst 33342. Emissions from the FITC, PE, and Hoechst 33342 were observed in the 496–537 nm, 652–690 nm, and 430–461 nm wavelengths, respectively. The brightness of the cell bodies and nuclei was measured using the Video TesT-Morphology software program (Video TesT, St. Petersburg, Russia). The immune-positive signal was analyzed if it was more than 300% of the background. Some slices were stained using the same protocol, but without the primary antibodies for the negative control. No traces of nonspecific immunoreactivity were observed.

Hippocampus cells were analyzed in a 400 μm wide section of the CA1 including layers st.oriens, st.pyramidale, and st.radiatum-moleculare (not divided on separated st.moleculare, lacunosum, and radiatum). The number of NeuN-positive neurons, GFAP-positive astrocytes, and Iba1-positive microglial cells were counted at the same field of vision for each brain slice. Besides, the total area of the immune-positive (more than 300% of the background level) structures per the analyzed area was also analyzed. The number of the immune-positive cells showed changes in the number of the neuronal and glial cells.

### 2.6. Electron Microscopy

On P5 and P20, the ultrastructure of the dorsal hippocampus (the same area as we used in the immunofluorescent analysis, *n =* 2 animals per group) was analyzed in PHHC and control pups. After perfusion (1% of glutaraldehyde, 1% formaldehyde in 0.1 M PBS, pH 7.4), brain tissue was fixed in 1% OsO_4_, stained with uranyl acetate, dehydrated, and embedded in Araldite following the protocol described previously [20]. Ultra-thin sections of 500Å thickness were made using an LKB-III ultramicrotome (LKB, Stockholm, Sweden) and analyzed using an FEI Tecnai Spirit V2 transmission electron microscope (FEI, Hillsboro, OR, USA). To evaluate the viability of neurons, the structural features of hippocampus cells were analyzed.

### 2.7. Cytokine Assay

For this assay, the left and the right hippocampus from the pups on P5 were pooled, while the left hippocampus was only used on P20. The tissue of the hippocampus was homogenized in 1:3 (*w/v*) 0.001 M PBS (pH 7.4). The homogenate was centrifuged at 16000× *g* for 20 min at 4 °C, and the supernatant was used in the assays. Tumor necrosis factor alpha (TNF-α), IL-1β, and interleukin-6 (IL-6) levels in the hippocampus were quantified by rat high-sensitivity enzyme-linked immunosorbent assays (ELISA) with commercially available kits, as per instructions provided by the manufacturer (R&D Systems, Minneapolis, MN, USA). The levels of interleukin-10 (IL-10) were analyzed using the ELISA kit (Cytokine Ltd., St. Petersburg, Russia), carefully following the manufacturer’s instructions. The content of these cytokines was measured through an optical densitometry at 450 nm in an ELx800 microplate reader (BioTek Instuments, Winooski, VT, USA).

### 2.8. Western Blot Analysis

For this assay, the left and the right hippocampus from the pups on P5 were pooled, while the right hippocampus was only used on P20. Brain tissues were homogenized on ice 1:2 (*w/v*) in a 0.001 M PBS buffer (pH 7.4). Tissue homogenates were then centrifuged at 16000x *g* for 20 min at 4 °C, and supernatants were collected into fresh tubes. Protein determinations in the homogenates were performed according to the Bradford method using bovine serum albumin as the standard. For the Western blot run, equal amounts of protein (50–80 μg as recommended for each antibody) for each sample were separated by electrophoresis using a 10% sodium dodecyl sulfate-polyacrylamide gel (Bio-Rad, Hercules, CA, USA). Proteins were transferred onto polyvinylidene difluoride (PVDF) membranes using a Trans-Blot^®^ Turbo™ system (Bio-Rad, Hercules, CA, USA). These PVDF membranes were blocked with 2% BSA (AppliChem GmbH, Darmstadt, Germany) in Tris-buffered saline plus 0.1% Tween-20 (Bio-Rad, Hercules, CA, USA) buffer (TBST) for 1.5 h at room temperature, and kept overnight at 4 °C with primary antibodies including rabbit monoclonal antibody against caspase-3 (9662S; Cell Signaling Technology, Danvers, MA, USA; dilution 1:1000), p38 MAPK (8690L; Cell Signaling Technology, Danvers, MA, USA; dilution 1:1000), GAPDH (2118L; Cell Signaling Technology, Danvers, MA, USA; dilution 1:1000), and mouse monoclonal antibody against phospho-p38 MAPK (9216L; Cell Signaling Technology, Danvers, MA, USA; dilution 1:1000). On the next day, blots were washed three times in TBST and incubated for 1.5 h with a secondary antibody, goat anti-rabbit, or anti-mouse Ig peroxidase conjugated (#1706515 or #1706516, BioRad, Hercules, CA, USA) at a dilution of 1:3000. After three final washes for 15 min each with TBST, bands were detected using enhanced chemiluminescence (Clarity Western ECL Substrate; Bio-Rad, Hercules, CA, USA) with the ChemiDoc Touch™ imaging system (Bio-Rad, Hercules, CA, USA). Densitometric analysis of each protein was conducted using the Image Lab™ 5.2.1 software (Bio-Rad, Hercules, CA, USA). Based on the existing recommendations for normalization of the target protein content [21], the obtained data were normalized to the content of GAPDH, with total protein content in the gel determined using the stain-free technology (BioRad, Hercules, CA, USA) according to the manufacturer’s instruction.

### 2.9. Caspase-3 Activity

For this assay, the left and the right hippocampus from the pups on P5 were pooled, while the right hippocampus was only used on P20. Caspase-3 activity was assayed in 20 mM HEPES, containing 0.1% CHAPS, 2 mM EDTA, and 5 mM DTT (pH 7.4) using 4 mM synthetic peptide Ac-DEVD-pNA (N-acetyl-Asp-Glu-Val-Asp p-nitroanilide) as a substrate. Samples containing 90 µg of protein were incubated at 37 °C for 10 min; the reaction was initiated by adding the substrate, and the absorbance of the reaction mixture was recorded at 405 nm at 37 °C every 5 min for 25 min. The activity of caspase-3 was defined as micromoles of the reaction product pNA per min per mg protein.

### 2.10. Acetylcholinesterase Activity

For this assay, the left and the right hippocampus from the pups on P5 were pooled, while the left hippocampus was only used on P20. The tissue of the hippocampus was homogenized in 3 volumes (1:3, *w/v*) of 0.001 M PBS buffer (pH 7.4) and centrifuged at 16000× *g* for 20 min at 4 °C. The supernatant was used for the enzymatic acetylcholinesterase (AChE) analyses. AChE activity was determined according to the Ellman method [22], with some modifications [23]. Hydrolysis rates were measured at acetylthiocholine concentration of 9.6 mM in 130 μL assay solution with 200 mM Na-phosphate buffer (pH 7.5) and 0.39 mM 5,5′-dithiobis-(2-nitrobenzoic acid) (DTNB). Equal amounts of protein (1.5 μg) for each sample were added to the reaction mixture. Samples were incubated for 10.5 min at room temperature for development of the colored product and the reaction was terminated by addition of 3% sodium dodecyl sulfate (SDS). All samples were run in triplicate. The AChE assay was performed in the presence of 20 μM butyrylcholinesterase inhibitor ethopropazine hydrochloride (Sigma-Aldrich, St. Louis, MO, USA). The absorption of the colored product developed in the samples was measured at a wavelength of 405 nm. The calibration curve was plotted using cysteine as a standard, AChE activity being expressed as fold from control.

### 2.11. Statistical Analysis

The normality of the data was tested using the Shapiro–Wilk normality test. To verify the equality of variances, the Levene test was used. Statistical analysis was performed using the STATISTICA 10.0 software. Variance was analyzed by the Mann–Whitney *U*-test and Student’s *t*-test. Values of *p* ≤ 0.05 were considered statistically significant. Values that were normally distributed and compared by parametric statistical methods are presented as mean ± standard deviation (SD). The data, the distribution of which did not obey the normal law, and which were compared by nonparametric statistical methods, were presented as median (25th, 75th percentile), with whiskers—the minimum and maximum values.

## 3. Results

### 3.1. Light Microscopy

**Figure 1:**
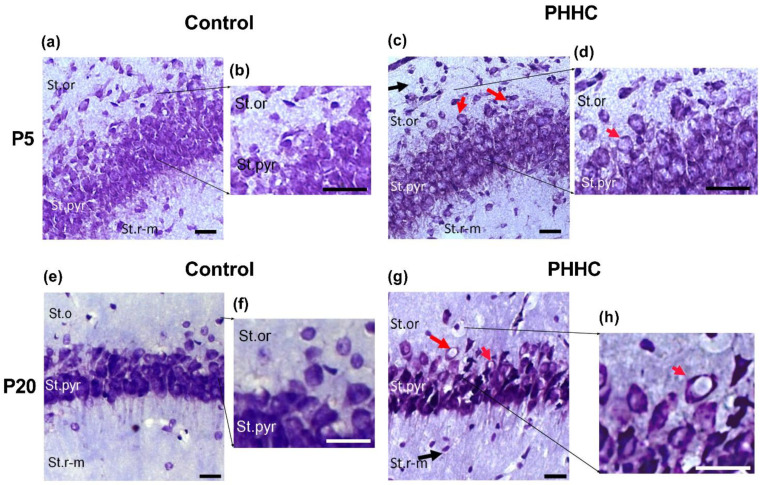
Microphotographs of the CA1 hippocampus tissue of the pups on P5 (**a**–**d**) and P20 (**e**–**h**) from control (**a**,**b**,**e**,**f**) and PHHC (**c**,**d**,**g**,**h**) groups. Scale (**a**,**c**,**e**,**g**): 30 μm; (**b**,**d**,**f**,**h**): 40 μm. Nissl staining. Red arrows: degenerating neurons; black arrows: glia cells. The layers of the CA1 area of the dorsal hippocampus are shown on the left part of microphotographs: St.pyr, stratum pyramidale; St.o, stratum oriens; St.r-m, stratum radiatum-moleculare; PHHC, prenatal hyperhomocysteinemia.

In early ontogenesis, a comparative study of the structural organization of the CA1 area of the dorsal hippocampus of pups subjected to PHHC was carried out using the Nissl method. Already on P5, stained sections in the pyramidal layer, and especially in the stratum oriens of the CA1 field of the hippocampus, showed signs of destruction of nerve cells, as compared with control animals (Figure 1a,b versus Figure 1c,d). Part of the projection neurons in the stratum pyramidale became swollen and lost their shape. Vacuoles and lysis of organelles appeared in the cytoplasm of these swollen neurons. A small number of altered interneurons with the same signs of cell destruction were found in the stratum oriens and the stratum radiatum-moleculare of the rat pups on P5 (Figure 1c,d). This type of degeneration is characteristic of cells in a state of chromatolysis. Numerous glial cells appeared in the neuropil of the hippocampus.

On P20, in rats from PHHC group, the number of neurons decreased compared to control animals. A large number of dying neurons surrounded by glial cells was observed in the neuropil (Figure 1g,h versus Figure 1e). Some loss of integrity was found in the pyramidal layer (Figure 1g) of P20 PHHC rats. In the stratum pyramidale of the CA1 area of the hippocampus, a significant number of swollen dying neurons were surrounded by glial cells. However, in the stratum oriens of the CA1 field of the hippocampus of P20 PHHC pups, the altered interneurons were less numerous than on P5. As in earlier age, the glial cells appeared to be numerous in the neuropil of the CA1 area of the dorsal hippocampus of P20 PHHC pups.

### 3.2. Electron Microscopy

**Figure 2:**
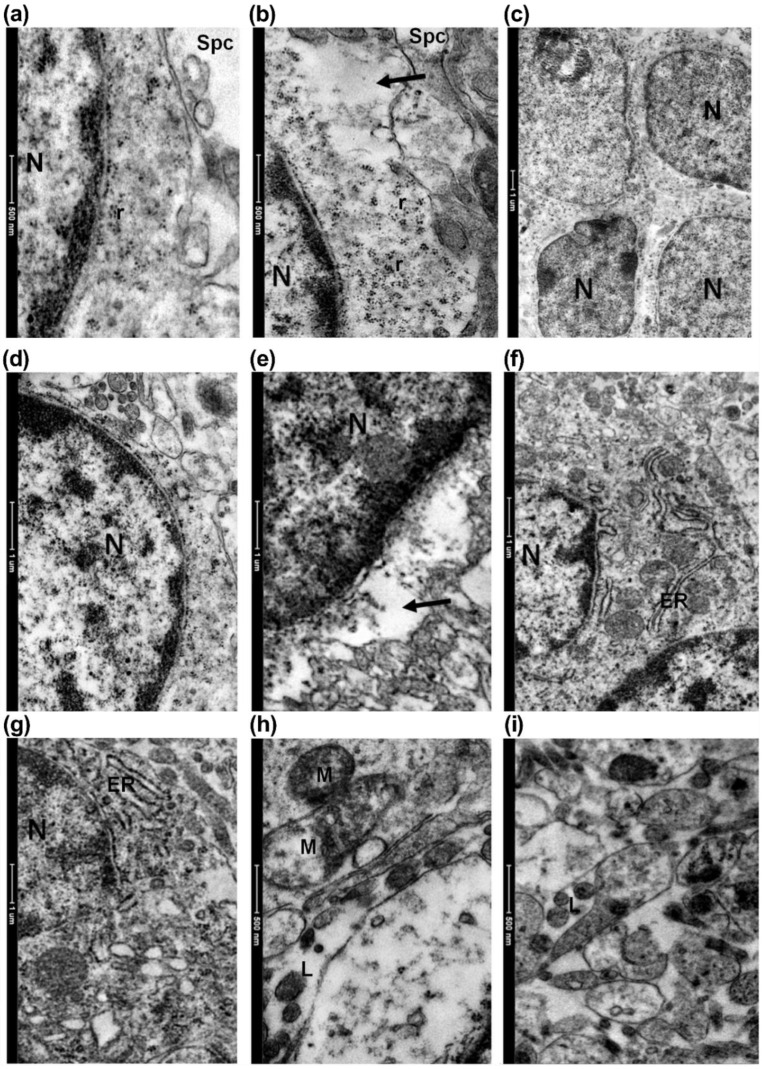
Ultrastructural images of the CA1 hippocampus of the pups on P5 (**a**–**c**) and P20 (**d**-**i**) in control (**a**,**d**) and after prenatal hyperhomocysteinemia (PHHC) (**b**,**c**,**e**–**i**). Electron microscopy. Spc, extracellular spaces; M, mitochondria; ER, endoplasmatic reticulum; G, Golgi apparatus; L, lysosomes; N, nucleus; r, clustered ribosomes. (**a**,**b**) Interneurons from the stratum oriens, (**c**) group of the glia cells from the stratum oriens, (**d**–**g**) projection pyramidal neurons from the stratum pyramidale, (**h**,**i**) fragment of the neuropil from the stratum pyramidale. In (**b**,**e**), cytoplasm of degenerating neurons (chromatolysis) with the area of the lysis of cell organelles (arrow) is shown.

The study of the ultrastructural organization of the hippocampus showed that, in both P5 and P20 PHHC rats, a significant number of swollen cells with lysis of the organelles in the cytoplasm (chromatolysis) appeared in the pyramidal layer of the CA1 field of the hippocampus (Figure 2e).

Unlike the control group, in PHHC pups, the swollen neurons with numerous single ribosomes and those combined into numerous polysomes were observed (Figure 2b). The endoplasmic reticulum (EPR) and Golgi apparatus was slightly expanded in some cells (Figure 2f), and numerous ribosomes were located on its surface. Often, numerous lysosomes were found in the cytoplasm of such swollen neurons, but they were solitary, rather than clustered. Lysosomes were found in PHHC pups more often than in control, not only in the cytoplasm of neurons, but also in dendrites (Figure 2h,i). Some neurons were swollen, with swollen and enlarged EPR channels. These signs are characteristic of cells in a state of chromatolysis (Figure 2b,e), which was observed in PHHC brain slices stained by the Nissl method (Figure 1b,e,f). Some neurons were surrounded by glial cells and overgrown glial processes. Besides the pyramidal layer, cell degeneration was found in the stratum oriens. Figure 2b shows an interneuron with a region of organelle lysis in the cytoplasm, surrounded by numerous ribosomes and polysomes. During this period of development, the glial cells were clustered surrounding degenerating neurons. The group of glial cells can be seen on Figure 2c. Such changes undergo cells in the stratum oriens (interneurons in Figure 2b) and in the stratum pyramidale (projection pyramidal cells in Figure 2e). The endoplasmic reticulum and Golgi apparatus in the cytoplasm of cells were slightly expanded (Figure 2f,g), in contrast to the neurons of the parietal cortex in rats with PHHC of the same age. The destruction of mitochondrial cristae was often observed in PHHC pups (Figure 2h) together with accumulation of lysosomes in the cytoplasm and processes of neurons (Figure 2i). Thus, the study of the structural and ultrastructural organization in rats after PHHC at P5-20 revealed degenerative changes in neurons: lysis of organelles in the cytoplasm, destruction of mitochondria, accumulation of lysosomes, and the appearance of a large number of glial cells.

### 3.3. PHHC-Induced Neuronal Cell Loss and Microglia Activation

**Figure 3:**
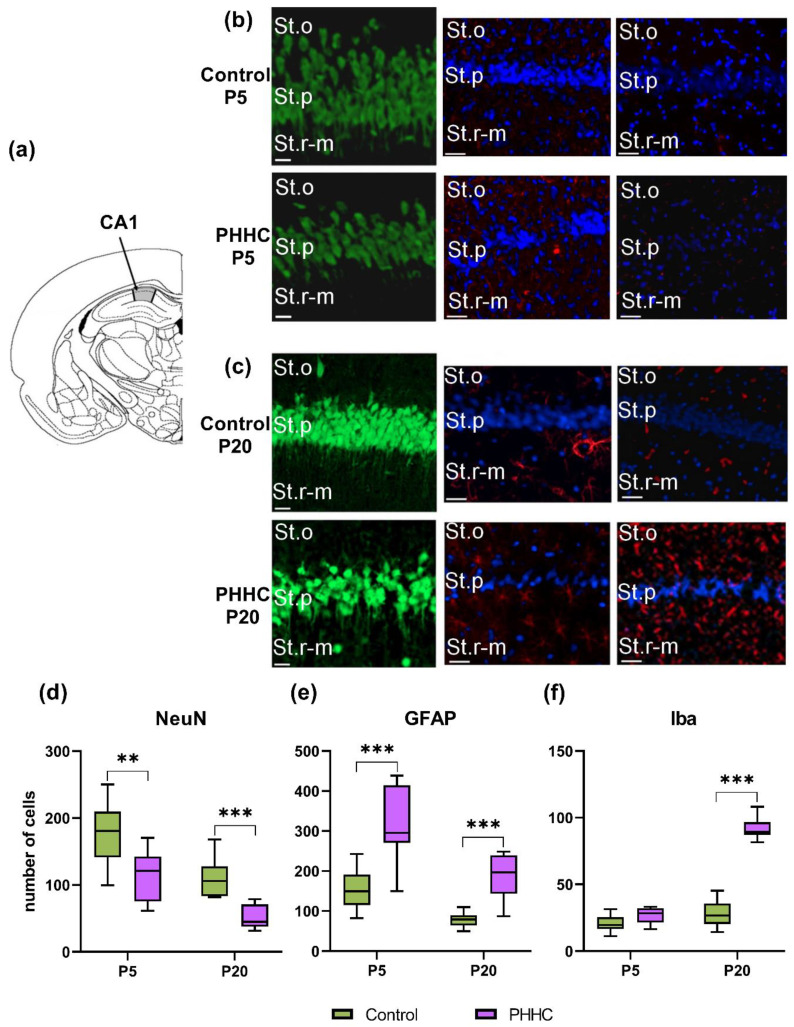
The effect of prenatal hyperhomocysteinemia (PHHC) on the number of neuronal and glial cells in the CA1 area of the dorsal hippocampus. (**a**) The scheme of the analyzed area in the hippocampus (gray shading). (**b**) Micrographs of the CA1 area of P5 control pups and pups subjected to PHHC. (**c**) Micrographs of the CA1 area of P20 control pups and pups subjected to PHHC. Immunohistochemical staining of neuronal marker protein NeuN (FITC, left), marker of astrocytes GFAP (PE, in the middle), or microglial marker Iba1 (PE, right). Cell nuclei were stained with non-specific nucleus dye DAPI (blue). Scale: 40 µm. The median number of cells in the hippocampus: NeuN-positive neurons (**d**), GFAP-positive (**e**), and Iba1-positive (**f**) glial cells in control pups (*n* = 8) and rats after PHHC (*n* = 8). Values are expressed as Me (25th, 75th percentile), whiskers—min-max. Significance of difference between the PHHC and control groups were determined by the Mann–Whitney U test (** *p* ≤ 0.01, *** *p* ≤ 0.001). St.p, stratum pyramidale; St.o, stratum oriens; St.r-m, stratum radiatum-moleculare.

The cellular composition of the CA1 area (Figure 3a) of the dorsal hippocampus in the pups of the control and PHHC groups is presented in Figure 3. The decrease in the number of viable hippocampus neurons expressing NeuN protein was observed in PHHC pups on P5 and P20 (Mann–Whitney *U*-test, *p* ≤ 0.01 for P5, *p* ≤ 0.001 for P20). In P5 PHHC pups, the number of neurons was 65.26% of the control level, and it decreased to 47.09% on P20 (Figure 3c). In addition, the glial reaction was observed in the CA1 area of the hippocampus in PHHC pups (Figure 3b). The number of glial cells was increased relative to the control. In P5 PHHC pups, the average astrocyte number was 2.09-fold higher than the control level (Mann–Whitney *U*-test, *p* ≤ 0.001; Figure 3d), while the number of microglia cells was the same as in control (Figure 3e). On P20, the glial reaction intensified: the amount of astroglia was 2.41-fold (Mann–Whitney *U*-test, *p* ≤ 0.001; Figure 3d) of the control, and the amount of microglia was 3.28-fold of the control level (Mann–Whitney *U*-test, *p* ≤ 0.001; Figure 3e).

### 3.4. Pro- and Anti-Inflammatory Cytokine Content

**Figure 4:**
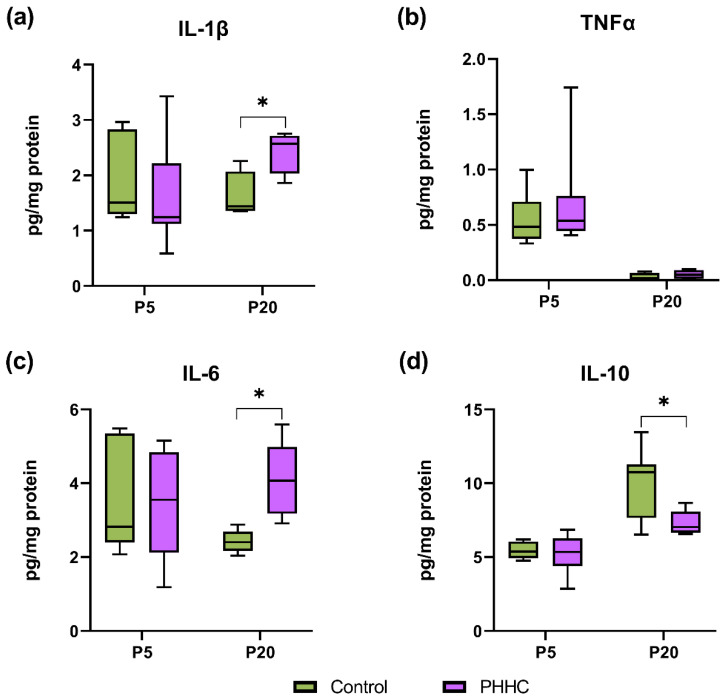
Effect of maternal hyperhomocysteinemia on the content of cytokines in the rat pup hippocampus. PHHC, prenatal hyperhomocysteinemia. (**a**) IL-1β content in the hippocampus of rats on P5 and P20 in the control (*n* = 7) and PHHC (*n* = 9) groups. (**b**) TNFα level in the hippocampus of rats on P5 and P20 in the control (*n* = 7) and PHHC (*n* = 9) groups. (**c**) IL-6 content in the hippocampus of rats on P5 and P20 in the control (*n* = 7) and PHHC (*n* = 9) groups. (**d**) IL-10 level in the hippocampus of rats on P5 and P20 in the control (*n* = 7) and PHHC (*n* = 9) groups. Data represent Me (25th, 75th percentile), whiskers—min-max. All samples were run in triplicate. Significance of difference between the PHHC and control groups were determined by the Student’s *t*-test (* *p* ≤ 0.05).

To investigate the proinflammatory reaction in rats after PHHC, we measured the levels of proinflammatory cytokines such as TNF-α, IL-1β, and IL-6 in the hippocampus homogenates. There was no significant difference in concentrations of cytokines between the PHHC and control groups of pups on P5. As shown in Figure 4a,c, the expression levels of IL-6 and IL-1β in the hippocampus of P20 pups from mothers with HHC during pregnancy were higher than those in the control group (Student’s *t*-test, *p* ≤ 0.05), demonstrating that PPHC can lead to the occurrence of inflammation and the release of proinflammatory cytokines.

Subsequently, we investigated the content of IL-10, which is one of the important anti-inflammatory cytokines, in the PHHC rats. On P20, the hippocampus homogenate level of IL-10 in the PHHC group of rat offspring was significantly decreased compared to the level in the control group (Student’s t-test, *p* ≤ 0.05) (Figure 4c).

### 3.5. Acetylcholinesterase Activity

**Figure 5:**
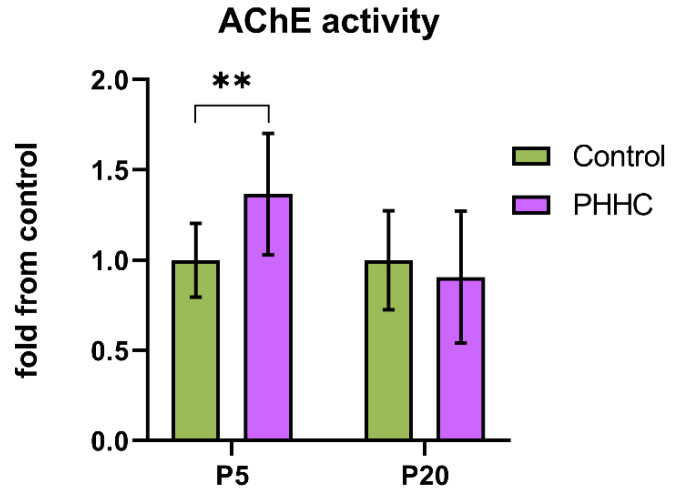
Effect of prenatal hyperhomocysteinemia (PHHC) on acetylcholinesterase activity in the hippocampus of the rat pups on P5 and P20. Data for each age are normalized to the appropriate control group for comparison and presented as mean ± SD, *n* = 10–19 for the control group, *n* = 9–19 for the PHHC group of animals performed in triplicate for activity. Significance of difference between the PHHC and control groups were determined by the Student’s *t*-test (** *p* ≤ 0.01).

An increase in AChE activity was observed in the hippocampus of pups in the PHHC group on P5 (1.37 ± 0.11 vs. 1.00 ± 0.06 in the control group; the Student’s *t*-test, *p* ≤ 0.01) (Figure 5). AChE activity on P20 did not differ between the control group and the pups after PHHC.

### 3.6. Caspase-3 Content and Activity

**Figure 6:**
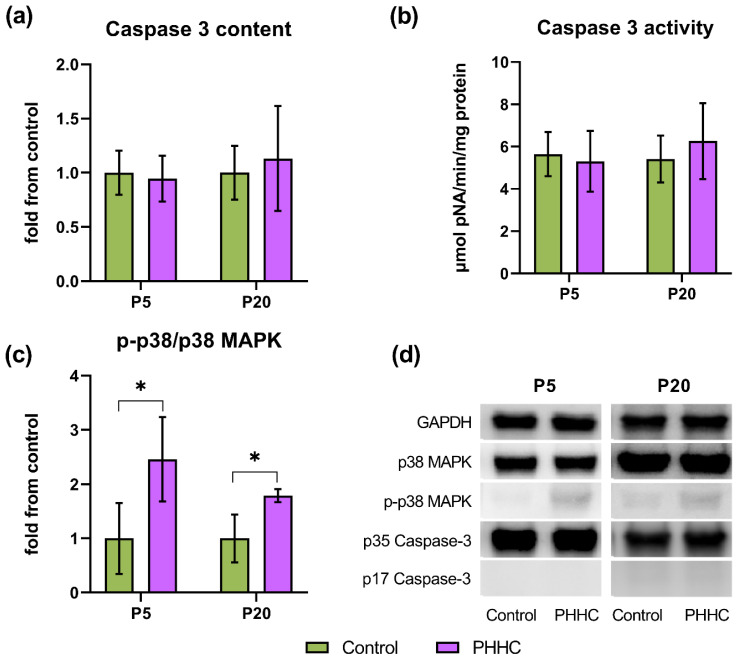
Effects of maternal hyperhomocysteinemia on caspase-3 and p38 MAPK in the hippocampus of the pups on P5 and P20. PHHC, prenatal hyperhomocysteinemia. (**a**) Band density of p35 caspase-3 content in the hippocampus of rats on P5 and P20 in the control (*n* = 12–13) and PHHC (*n* = 13–15) groups determined by densitometry analysis. (**b**) Caspase-3 activity in the hippocampus of the rats on P5 (*n* = 6–8) and P20 (*n* = 10) in the control and PHHC groups. Samples were run in duplicate. (**c**) The ratio of phospho-p38 (p-p38) and total p38 MAPK content in the hippocampus of the rats on P5 (*n* = 4–5) and P20 (*n* = 4–5) in the control and PHHC groups determined by densitometry analysis. (**d**) Representative Western blots are shown for each condition. Western blot data for each age are normalized to the appropriate control group for comparison. Data are presented as mean ± SD. Significance of difference between the PHHC and control groups were determined by the Student’s *t*-test (* *p* ≤ 0.05).

Despite the fact that in this work we used the same antibodies as in the analysis of the cortex, we did not detect the cleavage of procaspase-3 (p35) to its active form (p17) (Figure 6d). Moreover, there was no significant difference in content of procaspase-3 and the activity of this enzyme in the hippocampus of rats with PHHC and the control groups of pups on either P5, or P20 (Figure 6a,b). Procaspase-3 content and activity on P20 only showed a tendency to increase in the group of pups after PHHC.

### 3.7. p38 MAPK Content

The p38 MAPK phosphorylation in the hippocampus of PHHC rats increased on P5 (2.44-fold, the Mann–Whitney *U*-test) (Figure 6c), despite the fact that the total levels of p38 MAPK were not altered (Figure 6d). Furthermore, the ratio of phospho-p38 (p-p38) and total p38 MAPK content was also elevated in the PHHC group of pups on P20 (1.77-fold, the Mann–Whitney *U*-test *p* ≤ 0.05) (Figure 6c).

## 4. Discussion

To identify the possible causes of the behavioral changes observed previously in the rat offspring after PHHC [1,7], we investigated the glial cells and neurons in the hippocampus using immunofluorescence staining. Microglia are integral resident central nervous system immune cells [24,25] and excessive and prolonged activation of microglial cells can lead to a variety of pathological damages in the nervous tissue [26]. Microglia continually survey the brain parenchyma and upon activation are responsible for rapidly redirecting processes, phagocytosis, and augmenting inflammation via the production of reactive oxygen species and proinflammatory cytokines such as nitric oxide, arachidonic acid, TNF-α, and IL-1β, in turn increasing oxidative stress and leading to further neuronal cell death [27]. Several studies have shown that astrocytes can also respond to different stimuli including Hcy treatment, releasing such proinflammatory cytokines as TNF-α, IL-1β, and IL-6 [28,29]. It was demonstrated that an in vivo rat experimental model of mild HHC promoted an increase in the same inflammatory mediators in the cerebral cortex [30]. As for the prenatal model of HHC, daily methionine injections during gestation did not alter TNF-α and IL-6 levels in the whole brain of rat offspring on P21 [31]; however, the concentration of IL-1β was not determined. In previous studies, we observed higher levels of IL-1β and activated microglia and astroglia in the cortex of rats subjected to PHHC [18]. The results of present work have shown that maternal HHC increased both IL-6 and IL-1β levels in the hippocampus of rat pups on P20. IL-1β is an essential player in neuron-glia cross talk that has been demonstrated to mediate neuronal injury and potentiate excitotoxicity [32]. As with IL-1β, IL-6 is reported to alter NMDA receptor-mediated response and to enhance neurotoxicity [33]. Furthermore, the recent study of our research team has shown that cerebellar neurons from animals developed under conditions of maternal HHC were characterized by desensitization of NMDA receptors [34].

Elevation of Hcy level during chronic methionine administration to rats was found to decrease the plasma concentration of IL-10 but there was no effect on the expression of this anti-inflammatory cytokine in brain tissues [35]. The same authors showed that the IL-10 mRNA levels were manifestly downregulated in the brain of spontaneously hypertensive rats with elevated blood concentration of Hcy [36]. Another study demonstrated that HHC decreased TNF-α and increased IL-1β gene expression in the striatum of rats while in the cerebellum, expression of TNF-α, IL-1β, IL-10, and TGF-β was increased [37]. In the present work, we demonstrated for the first time that gestational HHC decreased IL-10 content in the hippocampus of rats on P20, which, together with glia activation and the increase in IL-6 and IL-1β levels, suggests stronger inflammatory response to stressful stimuli in this structure than in the cortex.

Our results show that PHHC enhances AChE activity in the hippocampus of rats on P5, suggesting a reduction in acetylcholine levels leading to a proinflammatory state. High AChE activity together with elevated cytokine content were observed under different stressful conditions including alcohol and LPS exposure in adult animals [38,39]. Chronic mild HHC was reported to enhance AChE activity together with IL-1β and IL-6 levels in the cerebral cortex and the hippocampus of adult rats [40,41]. It has also been shown that acetylcholine inhibits the release of the proinflammatory cytokines TNF, IL-1β, and IL-6 [42] and AChE inhibition reduces microglial production of TNF-α in a hypoxia model [43]. Furthermore, changes in choline metabolism and increase in the choline acetyltransferase protein level were reported in the hippocampus of mice offspring in response to maternal folate deficiency during pregnancy [4]. In our study, the time of the increase in AChE activity does not coincide with the period of the increased proinflammatory cytokines levels, but it coincides with the early development of astrogliosis in CA1 of hippocampus in PHHC pups. It has been shown that astrocytes are able to synthesize acetylcholine [44], which in turn downregulates the activation of microglia through the a7 nAChR [45]. It can be assumed that the increase in AChE activity in our case has a compensatory effect. The supposed decrease in the acetylcholine level leads microglia to a hyperactivation state and the development of persistent neuroinflammation and exacerbation of neurodegeneration, which we observed in the hippocampus of pups on P20. Thus, one can speculate that the increase in the proinflammatory cytokine levels in the hippocampus caused by maternal HHC can be partly associated with the enhancement of AChE activity, since this enzyme hydrolyzes acetylcholine, which is considered an anti-inflammatory molecule that can act by inhibiting the production of proinflammatory mediators [42].

Previous studies have well established the role of STAT3 and p38 MAPK in neuroinflammation [46,47,48,49]. STAT3 is one of key factors involved in many cytokine cascades, including IL-6, IL-10, and TNFα [50,51]. Moreover, the STAT3 in astrocytes is an essential step by which astrocytes realize a proinflammatory response in the central nervous system. Hcy has been reported to promote proliferation and activation of microglia through induction of STAT3 (tyr705) phosphorylation [48]. The anti-inflammatory effect of IL-10 has also been suggested to be mediated via activation of STAT3. In turn, elevated phosphorylation of p38 MAPK has been reported in chronic cerebral hypoperfusion and cell models that had suffered inflammatory damage [52], which further induces production of proinflammatory factors, including TNF-a [53,54,55]. Increased p38 MAPK phosphorylation was also associated with production of TNFα and IL-6 in the model of microglial polarization induced with LPS [56]. We have recently observed markedly induced levels of phosphorylated p38 MAPK in the rat pups cortex on P5, at the time that blood and brain Hcy levels were normalized after PHHC [18]. Consistently, the present study also showed that rat pups had over-phosphorylated p38 MAPK in the hippocampus after PHHC during the first month of postnatal life (on P5 and P20). Elevated expressions of inflammatory molecules may combine to have a contributing negative effect on neurons in the postnatal hippocampus. Several studies have shown that p38 MAPK phosphorylation increased in the hippocampus and the brain cortex under hypoxic conditions [57], and this p38 MAPK activation coincided with neuronal apoptosis and cognitive function deficit in young rats [58]. Central nervous system proinflammatory cytokine overproduction and release activated by p38 MAPK have been reported to promote neuronal damage [59,60].

Neurons and astroglial cells have been found to potentially accumulate Hcy [3,61,62]. In the rat offspring of mothers with a diet lacking methyl donors (vitamins B_12_, B_2_, folate, and choline) during pregnancy, the presence of Hcy in high concentrations was shown in specific regions of the brain, such as the cerebellum, the CA1 pyramidal layer of the hippocampus, the striatum, and the subventricular zone lining the lateral ventricle [3]. As previously reported, our model of PHHC also induced elevation of Hcy level in the serum and the brain of fetuses on the 20th day of prenatal development [17] and up to the third day after birth [18]. Consequently, there is reason to believe that increased serum Hcy levels in mothers could readily be transferred in utero and contribute to increased cell death in the brain of the offspring.

According to the published data, apoptosis activation during HHC in different types of cells could occur via either the external pathway through the action of extracellular signal on the membrane receptors, or the internal pathway associated with mitochondria destruction under oxidative stress conditions or inflammation [48,63]. PHHC was shown to induce the cleavage of DNA into oligonucleosome-length fragments and to elevate the levels of p53 mRNA, as well as to reduce Bcl-2 content in brain tissue of newborn pups [64]. These data are consistent with those of our studies that showed an increase of caspase-3 activity in the fetal brain [17]. According to postnatal effects, our previous results revealed that PHHC caused reduction in neurons in line with caspase-3 cleavage and activity in the cortex of rats suggesting apoptosis activation [18]. Like in the cortex, in the hippocampus of rats on P5 and P20, we found that the number of neurons (NeuN-positive cells) in PHHC pups was lower than in the control group. Nevertheless, changes associated with caspase activation were evident in the prefrontal cortex [18] but not in the hippocampus. Hcy was shown to activate not only the caspase-dependent signaling pathway, but also other mechanisms of cell death, which are realized through the release of AIF and EndoG from mitochondria [65,66]. Studies have found that maternal MTHFR deficiency during gestation resulted in increased apoptosis in the hippocampus of P21 mice offspring [4]. However, low level of folic acid during embryogenesis did not affect the number of TUNEL-positive cells in the hippocampus [4], while maternal diet lacking more methyl donors resulted in a substantial increase in cells that were positive for the specific antibody against single-stranded DNA, as well as for p53 [3]. Thus, it can be assumed that the presence of apoptosis may depend on the severity of HHC, as well as on brain region.

Due to the fact that Hcy and its metabolites have neurotoxic properties, numerous studies have described their effect on the nervous system in general and on individual cells, in particular. The most studied mechanisms of the neurotoxic action of Hcy include the development of oxidative stress under its influence [64,67,68] and excitotoxic effect due to structural similarity to glutamate [69,70]. However, recently, the essential role of HHC in epigenetic modifications associated with methylation reactions, primarily DNA methylation, has been actively studied [71,72,73,74,75]. Chronic elevation in Hcy level leads to a parallel increase in intracellular S-adenosyl-Hcy (SAH) with a strong inhibition of DNA methyltransferases. SAH-mediated DNA hypomethylation and associated changes in the expression of specific genes and chromatin structure may provide new hypotheses for the pathogenesis of diseases associated with HHC. The functional consequences of the decreased methylation ability are significant and include demyelination of the central nervous system [76], decreased synthesis of neurotransmitters [77], changes in membrane phospholipid composition and membrane fluidity [78], impaired gene expression [75], and cell differentiation [72].

Our results showed that PHHC caused biochemical alterations and, consequently, cell number changed. In order to complete these results, we performed a transmission electron microscopical analysis that showed that the impairments caused by maternal HHC led, at least in part, to the ultrastructural alterations. PHHC induced an appearance of swollen neurons with hypertrophically enlarged EPR channels and accumulation of ribosomes in the cytoplasm, which was consistent with the reports by other authors [6,79,80]. In adult animals, HHC was shown to induce some mitochondria dysfunction and ultrastructural changes in the hippocampus and cortex [81]. The treatment of adult rats with Hcy led to significant reduction in the number of neuronal cells, degradation of their synapses and dendritic spines, indicating Hcy-associated neurodegeneration [82]. Furthermore, methionine-enriched diet was shown to alter the volume of adult rat CA1 hippocampus, astroglial activation, and swelling of soma and nuclei of neurons [83]. In the present study, no neuronal death by hyperchromatosis type with wrinkling of cell bodies and their processes with electron-dense cytoplasm was found in the hippocampus of PHHC rats compared to the parietal cortex [18]. The cells demonstrating chromatolysis in the CA1 area of the hippocampus of PHHC rats were less numerous than in the brain cortex. The hypertrophically enlarged EPR cisterns were more numerous in cortical neurons than in the hippocampus. As the hypertrophy of the EPR and Golgi apparatus might lead to the changes in cell turgor and appearance of the unstained cytoplasm areas, the correlation of these two structural changes might be predictable. The accumulation of ribosomes in the cytoplasm, especially their polysomic forms, is known to be associated with neuronal viability and might suggest subsequent neuronal death [84]. In some experimental models of axotomy and neurotoxicity, it was shown that chromatolysis might be a reversible dysfunction of protein synthesis machinery, indicating the activation of neuroprotective mechanisms leading to neuronal function recovery [85]. Therefore, changes in the ribosome number and distribution observed in cortical and hippocampal neurons of PHHC pups might be associated with restorable changes of EPR rather than neuronal death. The lysis of the cell organelles is an unspecific feature of cell destruction [86] and was reported in different pathological models including PHHC [6]. The increase in the number of glial cells and their processes, as well as the numerous lysosomes in neuronal cells indicate the role of neuroinflammation in hippocampal tissue of PHHC pups. Comparative analysis of changes in the hippocampus and the cortex suggests the involvement of various mechanisms of neuronal cell death, as well as specific features of the development of these brain regions leading to differences in their vulnerability to the PHHC.

## 5. Conclusions

In summary, our findings demonstrated that maternal HHC seems to negatively affect the cytoarchitecture and function of the hippocampus of infant rats. Histological and biochemical examination of the hippocampus revealed alterations in the ultrastructure of neurons, as well as their loss without activation of caspase-3. The data obtained might suggest the involvement of various mechanisms of neuronal cell death in the hippocampus and cortical tissue leading to differences in their vulnerability to PHHC. AChE activity was also altered, suggesting that PHHC may promote neuroinflamatory processes. Neuroinflammation, reflected by changes in the level of proinflammatory (IL-1β and IL-6) and anti-inflammatory (IL-10) cytokines, as well as the activation of astrocytes and microglia in the hippocampus of affected offspring, may be some of the mechanisms for the behavioral disorders resulting from prenatal exposure to maternal HHC.

## Data Availability

The data presented in this study are available on request from the corresponding author.

## Abbreviations

AChE	acetylcholinesterase
EPR	endoplasmatic reticulum
GAPDH	glyceraldehyde 3-phosphate dehydrogenase
GFAP	glial fibrillary acidic protein
Hcy	homocysteine
HHC	hyperhomocysteinemia
Iba1	ionized calcium-binding adapter molecule
IL-1β	interleukin-1β
IL-6	interleukin-6
IL-10	interleukin-10
MAPK	mitogen-activated protein kinase
Mthfr	methylenetetrahydrofolate reductase
NeuN	neuronal nuclear marker protein
PHHC	prenatal hyperhomocysteinemia
SAH	S-adenosyl homocysteine
SDS	sodium dodecyl sulfate
STAT3	signal transducer and activator of transcription 3
TNFα	tumor necrosis factor alpha
